# Production and Selectivity of Key Fusarubins from *Fusarium solani* due to Media Composition

**DOI:** 10.3390/toxins13060376

**Published:** 2021-05-25

**Authors:** Sebastian Birkedal Kristensen, Tobias Bruun Pedersen, Mikkel Rank Nielsen, Reinhard Wimmer, Jens Muff, Jens Laurids Sørensen

**Affiliations:** 1 Department of Chemistry and Bioscience, Aalborg University, 6700 Esbjerg, Denmark; sbk@bio.aau.dk (S.B.K.); tbp@bio.aau.dk (T.B.P.); mrn@bio.aau.dk (M.R.N.); jm@bio.aau.dk (J.M.); 2 Department of Chemistry and Bioscience, Aalborg University, 9220 Aalborg, Denmark; rw@bio.aau.dk

**Keywords:** *Fusarium*, secondary metabolites, polyketides, mycotoxins, pigments, quinones, fusarubins, *PKS3*, natural products

## Abstract

Natural products display a large structural variation and different uses within a broad spectrum of industries. In this study, we investigate the influence of carbohydrates and nitrogen sources on the production and selectivity of production of four different polyketides produced by *Fusarium solani*, fusarubin, javanicin, bostrycoidin and anhydrofusarubin. We introduce four different carbohydrates and two types of nitrogen sources. Hereafter, a full factorial design was applied using combinations of three levels of sucrose and three levels of the two types of nitrogen. Each combination displayed different selectivity and production yields for all the compounds of interest. Response surface design was utilized to investigate possible maximum yields for the surrounding combinations of media. It was also shown that the maximum yields were not always the ones illustrating high selectivity, which is an important factor for making purification steps easier. We visualized the production over time for one of the media types, illustrating high yields and selectivity.

## 1. Introduction

Fungi produce a plethora of different polyketides, some of which have beneficial pharmaceutical properties, including anti-tumor (e.g., aspergiolide A [1]), immunosuppressants (cyclosporin [2]), cholesterol lowering (e.g., lovastatin [3,4]) and antibacterial (e.g., penicillin [5]). Other compounds have mycotoxin effects on raw feed for livestock, crops and also humans [6,7,8] and are potent carcinogens (e.g., aflatoxin [6] and fumonisins [9]) or estrogenic (e.g., zearalenone [10], fusarin C [11] and fusarielins [12]).

**Figure 1:**
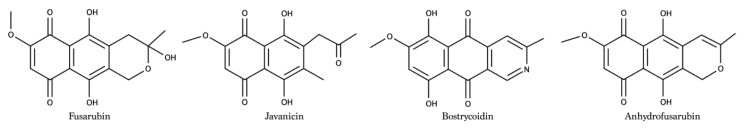
Structures of the four selected polyketide pigments produced by PKS3 in *Fusarium solani*.

In fungi, pigmentation could also primarily be attributed to polyketides [13]. The genus *Fusarium* is collectively able to produce the three polyketide pigments aurofusarin, bikaverin and fusarubins (incl. bostrycoidin, javanicin, Figure 1). Most *Fusarium* species use aurofusarin and bikaverin as mycelial pigmentation and fusarubins in perithecia [14,15]. However, in *F. solani* and closely related species, fusarubins are produced during mycelial growth, while a yet unidentified pigment is produced during sexual development in perithecia. The fusarubin gene cluster, *PKS3* (also described as *fsr* and *pgl)*, is found in all genome sequenced Fusaria, and is a non-reducing iterative type I PKS [16], which has been assigned earlier to produce 8-O-methylfusarubin in *F. fujikuroi* [14] and also the aza-anthraquinone bostrycoidin in *F. graminearum*. Bostrycoidin and fusarubin are believed to be synthesized from the same precursor, by the formation of a C14 heptaketide-aldehyde as the common intermediate [17,18]. Both fusarubin, bostrycoidin and many of the other intermediates in the biosynthetic pathway have proved to possess biological activity, including antimicrobial and antitubercular properties [19], antifungal activity [15], anticancer properties [20] as well as prominent activity toward *Staphylococcus aureus, Escherichia coli, Pseudomonas aeruginosa* and *Bacillus megaterium*. They also display potential cytotoxic properties toward human leukemia cells [21,22].

The cytotoxic and antimicrobial effect can be explained by the stimulation of cellular respiration and membrane NADH oxidation of bacteria [23,24]. The cytotoxic mechanism of naphthoquinones has also been described by Medentsev et al. [25,26] and Futuro et al. [27], where the flavoprotein-catalyzed redox cycle of the naphthoquinone creates a cascade of free radicals and superoxide molecules and other biochemical processes, which can damage DNA or RNA. The redox activity of natural occurring quinones was recently determined in silico, and several members of the fusarubin class were among the most reactive [28].

The fungal polyketides and secondary metabolites are often produced together with many different compounds. Therefore, it can prove challenging to produce pure compounds for use in the industry. The heterologous expression of genes in, e.g., yeast or bacteria is often used to produce fungal compounds in a different organism [29,30]. Pedersen et al. showed a heterologous expression of core genes in *F. solani* for a high-concentration production of pigments in *Saccharomyces cerevisiae* [31]. The yield of the compound bostrycoidin was not higher than 2.2 mg/L, which is low when compared to the metabolite extracted from the fungi, e.g., 21 mg/L by Kurobane et al. [32]. They have also shown production levels of 189 mg/L of fusarubin and 45 mg/L of the metabolite javanicin. Because the quinones and polyketides in general are produced as secondary metabolites depending on regulation factors, such as pH, carbohydrates, etc., it will be possible to alter the production portfolio of the fungi depending on the growth conditions and media composition. This has previously been demonstrated in a study by Sørensen et al., where the influence of both a different yeast extract [33] and carbohydrates [34] has been shown to influence the production of secondary metabolites. Medentsev and Akimenko discovered the influence of pH on the production of pigments in fungi, where both high and low pH provided inhibitory effects on the growth rate of the fungi. It was also shown that the nature of the pigments produced was different [25].

The aim of this study is to investigate the influence of medium composition on the production of the polyketides, fusarubin, javanicin, anhydrofusarubin and bostrycoidin by the fungus *F. solani*.

## 2. Results and Discussion

**Figure 2:**
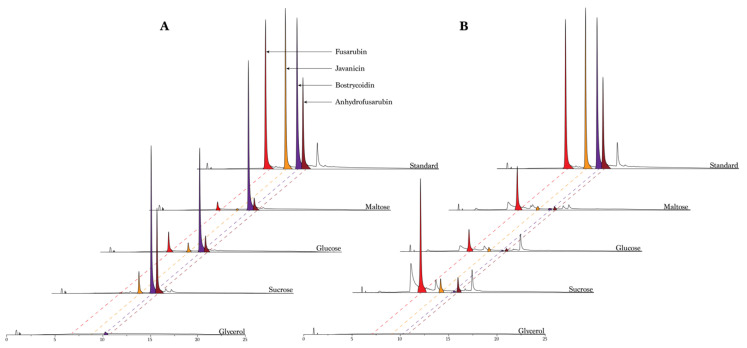
The effect of carbon and nitrogen sources on the production of PKS3-derived polyketides. All media contained 50 g/L carbohydrate and have been cultivated for seven days. (**A**) Chromatograms from samples where the media contained ammonium tartrate, fusarubin (red), javanicin (orange), bostrycoidin (purple) and anhydrofusarubin (red-brown) are indicated on the standard and by the dotted lines. (**B**) Chromatograms from samples where the media contained sodium nitrate. Fusarubin (red), Javanicin (orange), bostrycoidin (purple) and anhydrofusarubin (red-brown) is indicated by the dotted lines and color. Standard concentrations for both (**A**,**B**) are 53 mg/L for fusarubin, 39 mg/L for javanicin and bostrycoidin, and 19 mg/L for anhydrofusarubin.

As the *Fusarium solani* species complex is responsible for producing several compounds from the fusarubin biosynthetic pathway, it is interesting to investigate whether it is possible to enhance the production of these valuable compounds and if to produce compounds more selectively by varying their main substances and their concentrations in the growth medium. We started by investigating the effect of four different types of carbohydrates (maltose, glucose, sucrose and glycerol) and two different types of nitrogen sources (ammonium tartrate and sodium nitrate) on pigment production. The resulting chromatograms revealed a significant difference in the peak heights and number of peaks, depending on the carbohydrate type and nitrogen source (Figure 2). Fusarubin was only produced in small amounts in the samples including ammonium tartrate, except when using glucose as the carbohydrate source. The opposite behavior was observed for bostrycoidin, which was abundant when the fungus was grown in media containing ammonium tartrate, and when cultivated in media containing glycerol, where no compounds were produced in any significance. In a similar way, bostrycoidin was almost absent when the fungus grew in media containing sodium nitrate. Javanicin was produced in all different media, besides the glycerol media, with the highest peaks originating from the media with a combination of sucrose and ammonium tartrate. The media type containing glycerol showed no indication of producing fusarubins besides small quantities of bostrycoidin, when combined with ammonium tartrate. The media did not seem to favor the growth of the fungi, with no visible mycelium formation.

**Figure 3:**
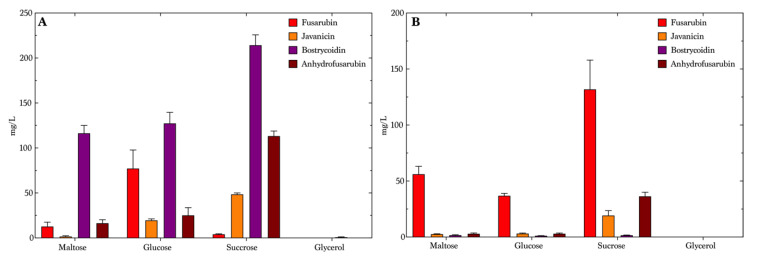
Mean concentrations (*n* = 4) of the four compounds of interest. Error bars indicate the standard error of mean in the media containing different carbohydrates and a nitrogen source. Cultivation period was seven days. (**A**) Ammonium tartrate, (**B**) Sodium nitrate.

We decided to use the same mass of carbohydrates, neglecting the fact that the carbohydrates used in this study do not have the same molar mass, and hence the molarity will be higher for carbohydrates with a lower molar mass. However, this did not influence our study, as the two selected disaccharides, sucrose and maltose, stimulated a higher production of the pigments on both sodium nitrate and ammonium tartrate (Figure 3 and Tables S1–S3). The optimal medium for bostrycoidin production was maltose combined with ammonium tartrate, yielding a mean concentration of 116 mg/L (Figure 3A), but the media types containing sucrose and glycose produced a mean concentration of 214 mg/L and 127 mg/L, respectively, though showing less selectivity, whereas the maltose media was chosen to be optimal for the production of bostrycoidin. Glycerol resulted in the lowest bostrycoidin concentrations of all media containing ammonium tartrate, no higher than 0.89 mg/L. The sucrose and ammonium tartrate combination was most favorable for anhydrofusarubin production, yielding 113 mg/L in average. This medium, however, did not produce much fusarubin. In the glycerol medium, fusarubin was produced with a mean concentration of 77 mg/L, and javanicin and anhydrofusarubin yielded 20 mg/L and 25 mg/L, respectively. In the medium containing sodium nitrate (Figure 3B), the production clearly favored fusarubin, with a yield of 56 mg/L in maltose medium, 37 mg/L in glucose medium and a mean concentration of 132 mg/L in the sucrose medium. Sucrose also yielded the highest production of javanicin (19 mg/L) and anhydrofusarubin (36 mg/L) in media with nitrate as the source of nitrogen. Bostrycoidin was produced only in minor quantities on glucose (1.2 mg/L) and on maltose (1.7 mg/L). No compounds were detectable in the glycerol medium. Except for fusarubin, the mean concentrations in the medium containing sodium nitrate were lower than the mean concentrations seen in the medium composed with ammonium tartrate.

The media containing sucrose was selected for further investigation, due to the variety of the difference in produced compounds and the high overall concentrations. We omitted maltose media despite the high production of bostrycoidin, because they already showed selectivity and high production yields.

**Figure 4:**
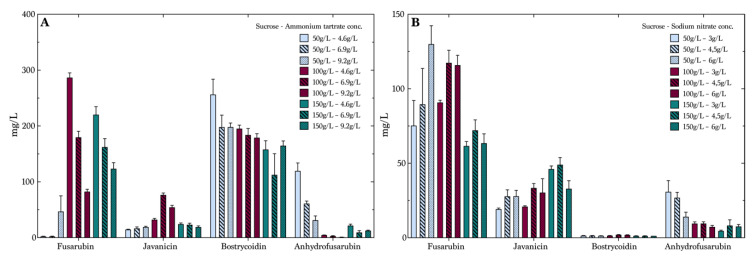
The effect of sucrose and nitrogen source concentrations on polyketide production. Average polyketide/product concentrations and standard error of mean (*n* = 4); cultivation period was seven days. (**A**) Media containing ammonium tartrate and (**B**) Media composed with sodium nitrate.

To optimize production on sucrose, we used a full factorial design of the experiments including three factors: carbohydrate concentration, nitrogen concentration and nitrogen source. The carbohydrate concentrations were 50 g/L, 100 g/L and 150 g/L and the concentration of nitrogen varied from 4.6 g/to 6.9 g/L and 9.2 g/L for the media containing ammonium tartrate. The second nitrogen source, sodium nitrate, had the following concentration levels: 3 g/L, 4.5 g/L and 6 g/L. As expected, the variation of concentration of the four target compounds was quite significant, indicating the possibility of tailoring a medium for the production of specific compounds. Due to fermentation in liquid media, we expected to see variations in the yields from experiment to experiment, larger than those of the experiments performed with fermentation in solid phases [35,36,37,38]. The variations and deviations between experiments when comparing the first experiment, illustrated in Figure 3A,B, and the second experiment, visualized in Figure 4A,B, are seen as natural variations in growth patterns due to, e.g., global regulation factors. The average values including the SD and SEM of the two experiments overlap and, therefore, cannot be determined as significant. Fusarubin was produced in low concentrations (Figure 4A) in the media composed of 50 g/L sucrose and ammonium tartrate, with an increase from 2.5 mg/L at an ammonium tartrate concentration of 4.6 g/L to 47 mg/L with an ammonium tartrate concentration of 9.2 g/L. However, the data showed that increasing sucrose and decreasing ammonium tartrate improved fusarubin production. Here, the 100 g/L sucrose and 4.6 g/L ammonium tartrate combination resulted in the highest production, of 287 mg/L, which was substantially higher than the previously described productions of fusarubin [32]. Furthermore, the effect plots showed that sucrose was the most important factor combined with the nitrogen concentration (Figures S1–S6). The remaining combinations of sucrose and ammonium tartrate decreased fusarubin production when ammonium tartrate levels were increased. Different tendencies were seen in the production of javanicin, even though the concentration levels were lower than for fusarubin. It was seen that a sucrose concentration of 100 g/L and an ammonium tartrate concentration of 6.9 g/L yielded the highest average concentration, of 77 mg/L. The production of bostrycoidin was overall stable, and high yields were observed in all of the samples. However, a slight decrease was seen with increasing sucrose and ammonium tartrate levels, as the lowest levels yielded the highest production. Anhydrofusarubin showed opposite tendencies of fusarubin, as it was seen that the 50 g/L and 4.6 g/L combination yielded the highest concentrations. This could indicate a correlation between the compounds, as a conversion might happen from one compound to the other. This has, however, not been investigated further. Figure 4B shows the average concentrations of the compounds in the sucrose media containing sodium nitrate. Here, fusarubin production displayed a different pattern than ammonium tartrate. There were indications that the production was slightly favored by a lower concentration of sucrose and a high nitrate concentration, with the production reaching 130 mg/L.

Javanicin production was optimal at the highest level of sucrose and the medium level of sodium nitrate, yielding 33 mg/L. It was noticeable that bostrycoidin was produced in significantly low levels compared to the first media containing ammonium tartrate, with a maximum average value of 2 mg/L. This was due to the need of a free ammonium to undergo the nonenzymatic oxidization producing bostrycoidin, as described by Kurobane et al. in 1980 [32]. Anhydrofusarubin was also produced in the highest quantities in the medium with the lowest level of sucrose and sodium nitrate. The data for individual compounds are presented in Figure S7 in the Supplementary Material.

**Figure 5:**
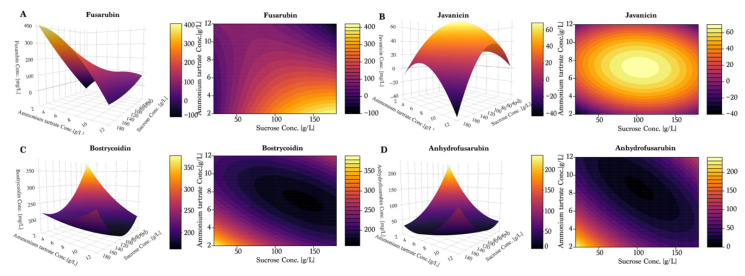
Response surface models and contour plots for predicted and measured concentrations of media composed of ammonium tartrate and sucrose. Cultivation time was seven days for the fungi used for measurements. (**A**) Fusarubin, (**B**) Javanicin, (**C**) Bostrycoidin and (**D**) Anhydrofusarubin.

**Figure 6:**
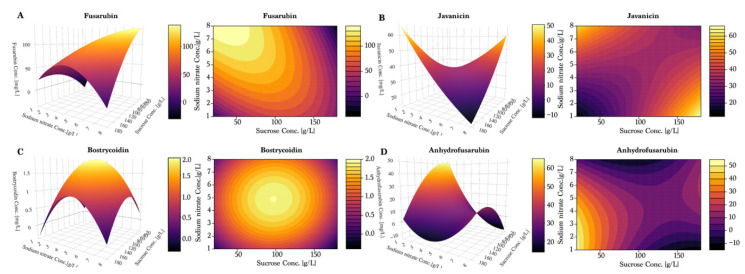
Response surface models and contour plots for predicted and measured concentrations of media composed of sucrose and sodium nitrate. Cultivation time was seven days for the fungi used for measurements. (**A**) Fusarubin, (**B**) Javanicin, (**C**) Bostrycoidin and (**D**) Anhydrofusarubin.

To further investigate the observed tendencies, a response surface design was prepared using the Box–Wilson central composite design, and can be seen in Figure 5 and Figure 6. Once again, fusarubin (Figure 5A) showed the highest production in a combination of high sucrose (180 g/L) and low ammonium tartrate, yielding up to 400 mg/L. Interestingly, the plot indicated that the optimum had not been found within these values. Javanicin (Figure 5B) displayed a hilltop shape, which indicated the presence of an optimum at 100 g/L of sucrose and 7–8 g/L of ammonium tartrate. For bostrycoidin (Figure 5C) and anhydrofusarubin (Figure 5D), the highest yields were achieved by lowering the sucrose and ammonium tartrate levels.

As observed in the previous experiments, fusarubin followed a linear trend of increasing yields under low sucrose and high nitrate (Figure 6A). However, the response surface plot also indicated that the optimal combination was not found within the tested values, even though a yield of 150 mg/L was achieved. The response surface of javanicin production (Figure 6B) suggested the existence of two distinct combinations of conditions for optimal production: for the first, a combination of high sucrose concentration and low sodium nitrate concentration; for the second, a low level of sucrose combined with a high concentration of sodium nitrate. This supports the hypothesis of javanicin being a co-metabolite and not a conversion compound like fusarubin or anhydrofusarubin.

In the response surface model, as measured previously, bostrycoidin (Figure 6C) production was low when nitrate was used as nitrogen source. The plot indicated that the maximum production was achieved with a combination of 100 g/L sucrose and 5 g/L sodium nitrate. Anhydrofusarubin tended to be produced in the highest quantities when composing the medium of low sucrose and sodium nitrate levels (Figure 6D). Furthermore, we observed an indication of a second optimum if sucrose and sodium nitrate were increased beyond the levels applied in our study.

**Figure 7:**
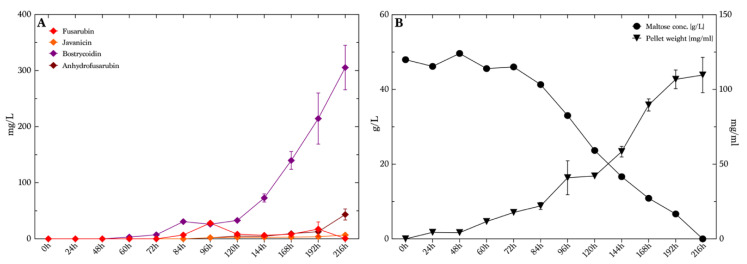
Production of pigments over time in bostrycoidin-favoring media conditions. (**A**) Average concentrations and standard deviation (*n* = 4) of the four quinones/polyketides produced over time, ranging from 0–216 h. (**B**) Carbohydrate consumption over time illustrated as circles referring to the left *y*-axis, and the mean dry weight of the cells (*n* = 4) in the samples referring to the right *y*-axis.

To investigate the isolated production of bostrycoidin, we chose to analyze the production levels at different time points during growth. For that, we used maltose medium with 50 g/L maltose and 4.6 g/L ammonium tartrate, as this illustrated high yields and good selectivity for bostrycoidin compared to the seven other combinations of the media found in the first part of the study in presence of ammonium tartrate. We decided to investigate bostrycoidin production, as this compound was produced in the overall highest concentrations throughout the whole study. The cultivation time was extended to 9 days (216 h) instead of the initial 7 days (168 h) which were used in previous experiments, based on earlier studies by Kurobane et al. [32], in order to investigate whether the production had reached a maximum or additional production could be achieved. The production of bostrycoidin was detected after 60 h with an average concentration of 3.2 mg/L (Figure 7A). The production slowly increased until 120 h, where the average yield was 33 mg/L. The average measured yields hereafter increased rapidly to the end of the experiments, reaching 305 mg/L, with no indication of declining production. The experiment was terminated at 216 h (9 days), when all maltose had been consumed in the medium (Figure 7B). The pellet weight also indicated that the fungi did not grow significantly between 192 h and 216 h, as the curve flattened out. The other three quinones were not present in high levels, as the highest average values measured were 28 mg/L of fusarubin at 96 h, 6.7 mg/L javanicin at 216 h and 43 mg/L of anhydrofusarubin at 216 h. The pigmentation of the media at every sampling time is illustrated in Figure S8 in the Supplementary Materials.

## 3. Conclusions

In this study, we present, how the production of fusarubin, javanicin, bostrycoidin and anhydrofusarubin in *F. solani* constitutively expressing the responsible gene cluster varies when grown on four different carbohydrates and two different nitrogen sources. It was shown that the media composed of maltose and ammonium tartrate yielded large amounts of bostrycoidin, with little to none of the other compounds. The sucrose medium was investigated further, as this medium produced all of the four compounds in different concentrations. A full factorial experimental design was initialized to investigate the effect of the different factors on the production of the compounds. It was concluded that the highest production of fusarubin was seen using the media containing ammonium tartrate, but the selectivity was not optimal, as bostrycoidin was also produced in large quantities. The optimal medium for producing fusarubin was established to be 50 g/L sucrose combined with 6 g/L sodium nitrate. For javanicin, the optimal medium was 150 g/L sucrose and 6 g/L of sodium nitrate. For anhydrofusarubin, the medium composed of 50 g/L of sucrose and 3 g/L of sodium nitrate indicated the highest yields when combining yields. These tendencies were validated by the response surface design models where indications of even higher theoretical yields could be seen. However, those media combinations remain to be tested. Because we used a constitutive promotor, we have assumed that the transcripts would be on similar levels in all cells, and then the difference in the production between the media could be assigned to the growth pattern of the fungi or the availability of substrates, such as acetyl-CoA and malonyl-CoA. Future experiments targeting this availability would enlighten this. The project also investigated maltose media and the production of bostrycoidin over a timeframe of 9 days. This led us to conclude that it was possible to exceed 300 mg/L of bostrycoidin for this medium type as well, with a high level of selectivity yielding little to no production of the three other compounds.

## 4. Materials and Methods

### 4.1. Fungal Strain and Macroconidia Isolation

*Fusarium solani* 77-13-4 OE::*fsr6* G418^R^ (Nielsen et al., 2019 [39]) was maintained on a PDA containing 100 µg/mL geneticin G418 at 28 °C. For inducing sporulation, a 100 mL CMC medium (15 g/L carboxymethylcellulose sodium salt, 1g/L NH_4_NO_3_, 1 g/L KH_2_PO_4_, 0.5 g/L MgSO_4_∙7H_2_O, 1 g/L bacto yeast extract) was inoculated with 5–10 agar squares from a week-old plate and incubated at 20 °C and 100 rpm for five days. The spores were harvested by pouring the culture through a sterile plastic syringe packed with glass wool removing agar and mycelium. The medium was removed by centrifugation at 10,000× *g* 4 °C for 15 min, discarding the supernatant. The spores were washed twice in 50 mL ice-cold sterile ultrapure water, followed by a final resuspension in 1 mL 10% glycerol, and stored at −80 °C.

### 4.2. Effect of Varying Carbon and Nitrogen Sources on Pigment Production

All media were initially based on Kurobane et al. [32] and modified following the base recipe according to our experiments. In the study investigating different carbohydrates, the OE::*fsr6 F. solani* strain was inoculated in media composed of: 50 g/L carbohydrate (maltose monohydrate (Merk KGaA, Darmstadt, Germany), glucose (Sigma-Aldrich, St. Louis, MO, USA), glycerol (Honeywell, Germany) or sucrose (MP biomedicals, Eschwege, Germany)), 4.6 g/L of ammonium tartrate (Acros organics, New Jersey, USA) or 3 g/L sodium nitrate (VWR, Herlev, Denmark), 1 g/L potassium dihydrogen phosphate (KH_2_PO_4_) (Merk KGaA, Darmstadt, Germany), 0.5 g/L magnesium sulfate heptahydrate (MgSO_4_∙7H_2_O) (VWR, Herlev, Denmark), 0.01 g/L ferrous sulfate heptahydrate (FeSO_4_∙7H_2_O) (Fluka, Sigma-Aldrich, St. Louis, MO, USA), 0.01 g/L sodium chloride (NaCl) (VWR, Herlev, Denmark) and 0.01 g/L calcium chloride (CaCl_2_) (Merk KGaA, Darmstadt, Germany), and 1 mL of the trace metal solution which contains 1.0 g ZnSO_4_∙7H_2_O (Merk KGaA, Darmstadt, Germany) and 0.5 g CuSO_4_∙5H_2_O (Acros organics, New Jersey, USA) in 100 mL MQ H_2_O. MQ H_2_O was produced by Synergy system UV (Millipore SAS, Molsheim, France). The pH was adjusted to 5.6 ± 0.2 for all media. OE::*fsr6* was inoculated containing 10,000 spores per 50 mL medium and incubated at 25 °C at 100 rpm for seven days, composing quadruplicates of all samples. The conditions correspond to a combination of the methods from Kurobane et al. [32] and Westphal et al. [40].

### 4.3. Production of Pigments in Response to Varying Concentrations of Sucrose

In the study, investigating different levels of carbohydrate and nitrogen source, the liquid media contained 50 g/L,100 g/L and 150 g/L of sucrose (MP biomedicals, Eschwege, Germany), and 4.6 g/L, 6.9 g/L and 9.2 g/L of ammonium tartrate (Acros organics, NJ, USA) or 3 g/L, 4.5 g/L and 6 g/l of sodium nitrate (VWR, Herlev, Denmark), and the rest of the media were composed as the base recipe. In all media, 250 mL were prepared using MQ H_2_O and autoclaved for 15 min at 121 °C after mixing. A capacity of 50 mL was then decanted into four Erlenmeyer flasks inoculated with 10,000 spores of OE::fsr6 and incubated at 25 °C at 100 rpm for seven days, constituting the quadruplicates of all experiments. The pH was adjusted to 5.6 ± 0.2 for all media.

### 4.4. Production of Bostrycoidin over Time

The media chosen to monitor bostrycoidin production over time contained 50 g/L maltose monohydrate (Merk KGaA, Darmstadt, Germany) and 4.6 g/L ammonium tartrate (Acros organics, New Jersey, USA). The rest of the medium was composed as the base recipe. The experiment was conducted with quadruplicates, where the 50,000 spores of the OE::*fsr6* strain were inoculated in 1000 mL Erlenmeyer flasks containing a 250 mL medium. The strain was cultivated at 25 °C at 100 rpm for 9 days.

Samples of 5 mL for pigment concentration determination, including 1 mL for carbohydrate concentration determination, were taken out after inoculation at 0 h, 24 h, 48 h and thereafter with 12 h intervals until 96 h. Later, the intervals were again of 24 h until 216 h or 9 days. The 12 h intervals were chosen because prior experiments had found the pigmentation starting phase to be around 48 h-96 h. The 5 mL samples were centrifuged at 5000× *g* for 5 min. The supernatant was extracted using LLE as described below. The pellet was weighed in wet weight and dried in an oven for 12 h at 105 °C, and then weighed again to determine the dry weight of the filaments in the sample. The 1 mL sample was filtered through 0.45 μm syringe filters (Frisenette, Knebel, Denmark), directly into 2 mL HPLC vials, and analyzed using HPLC-RID (Perkin Elmer Series 200, Perkin Elmer, MA, USA), coupled with a Perkin Elmer series 200 pump, Perkin Elmer series 200 autosampler, Perkin Elmer series 200a Refractive index detector, and a column oven. Injected volumes of 10 μL were analyzed at 85 °C, using an isocratic flow of 0.6 mL/min, of MQ H_2_O, through a Bio-Rad Aminex HPX-87P column (300 × 7.8 mm, Bio-Rad laboratories, USA).

### 4.5. Liquid-Liquid Extraction and HPLC-DAD Analysis of Secondary Metabolites from Liquid Media

The extraction procedure used in this study was initially based on Westphal et al.’s (2018) study on the production of aurofusarin [40], but the method was modified for the compounds and fungi extracted in this study. The growth media were filtrated through Miracloth (Millipore EMD, Billerica MA, USA). A sample size of a 10 mL medium was taken out for extraction and acidified by adding 0.7 mL 5 M HCl and shaken in 50 mL centrifuge tubes. Ten milliliters of chloroform (Sharlau, Sharlab S.L, Spain) were added to the media and shaken, and thereafter two phases were observed in the liquid and the color would primarily be in the water phase. We then added 6.7 mL methanol (VWR, Herlev, Denmark) and 6.7 mL 10 *w/v* % NaCl in MQ H_2_O solution to the mixture, which was shaken energetically. After the phases were separated, the majority of the color was observed in the organic phase. Most of the water was carefully decanted. Additionally, 6.7 mL methanol (VWR, Herlev, Denmark) and 6.7 mL 10 *w/v* % NaCl in MQ H_2_O solution were added to drive the last impurities to the water phase and all the pigment to the organic phase. The mixture was swirled and shaken calmly, and the phases were allowed to settle. The organic phase was collected by removing the water phase by pipetting. The organic phase was transferred to glass test tubes, and the chloroform was evaporated at 40 °C under N_2_ gas until complete dryness. The dried metabolites were resuspended in 2 mL methanol (VWR, Herlev, Denmark) and set for 10 min in an ultrasonic bath to ensure that all possible metabolites were dissolved. The dissolved metabolites were filtered through 0.22 μm syringe filters (Frisenette, Knebel, Denmark), directly into 2 mL HPLC vials.

Extracts were analyzed using HPLC-DAD on an Agilent 1260 infinity II HPLC system (Agilent, Santa Clara, USA), equipped with a 1260 vial sampler, a 1260 Quat. pump inclusive column oven set to 40 °C with a C6-phenyl column (Kinetex® 2.6 μm, 100 Å, 150 × 3 mm, Phenomenex, Torrance, CA, USA) and together with a guard column (UHPLC phenyl, 2.1mm ID column, Phenomenex, Torrance, CA, USA), 1260 DAD-HS measuring wavelengths at 280 nm, 310 nm and 495 nm, chosen—based on UV spectra yielded by pure compounds of fusarubin, javanicin and bostrycoidin, obtained by preparative HPLC and verified by NMR as described by Nielsen et al., 2019 [40]—and a 1260 RID unit.

The 0.5 mL/min gradient initiated at 80% solvent A (MQ H_2_O) and 20% solvent B (Acetonitrile, Hypersolve, VWR, Herlev, Denmark) added 50μL/L Trifluoroacetic acid (TFA Chromsolv, Sigma-Aldrich, St. Louis, MO, USA). The gradient increased linearly to 100% solvent B over 20 min, held for 5 min and then went back to the initial start composition, at 80% solvent A and 20% solvent B over a 5-min period, followed by 4 min post run.

### 4.6. Calibration and Standards for the Pigments

In order to be able to quantify the four target pigments, they were initially isolated from the extracts derived from OE::*fsr6*. This was performed by preparative HPLC (Thermo Scientific Ultimate 3000; Dionex Ultimate 3000 pump; Dionex Ultimate 3000 autosampler) coupled to a diode array detector (Dionex Ultimate 3000 DAD) and fraction collector (Dionex Ultimate 3000 fraction collector). The samples were fractionated using a C-18 column (Thermo Scientific, Hypersil Gold, 250 × 21.2 mm, 5 µm), injecting 0.5 mL per run, and applying a linear flow gradient of 10 mL/min. The gradient initiated at 80% solvent A (MQ H_2_O) and 20% solvent B (Acetonitrile, Hypersolve, VWR, Herlev, Denmark), both supplemented with 50 μL/L Trifluoroacetic acid (TFA Chromsolv, Sigma-Aldrich, St. Louis, MO, USA). The gradient increased linearly to 100% solvent B over 10 min, held for 5 min and then went back to the initial start composition, at 80% solvent A and 20% solvent B over a 3 min period. This gradient was continued for 5 min. The DAD was set to collect chromatograms at wavelengths 230 nm, 280 nm, 310 nm and 495 nm. The fraction collector (Dionex Ultimate 3000 fraction collector) was set to collect peaks in the chromatogram at 310 nm, with a peak start/end threshold of 10.00 mAu and peak slopes at ±0.500 mAu/s. The collected samples were analyzed using HPLC-DAD (Agilent 1260 infinity II HPLC system, Agilent, Santa Clara, USA), with the method and system described earlier, verifying the compounds fusarubin, javanicin, bostrycoidin and anhydrofusarubin. The fractions were dried at 40 °C under N_2_ gas until complete dryness and dry weight were noted. Stock solutions were made by resuspending samples in methanol (VWR, Herlev, Denmark), of which all concentrations were 5 g/L pigments, and then using previously determined compositions of the fractions to compose standard calibration solutions for fusarubin, javanicin, bostrycoidin and anhydrofusarubin. All calibration curves can be seen in Figure S9.

### 4.7. Statistical Analysis

All statistical analyses were performed using R studio (version 1.2.5033, Rstudio Inc.) and GraphPad prism 9 version 9.0.0 for macOS (GraphPad Software, San Diego, CA, USA).

## Supplementary Materials

The following are available online at https://www.mdpi.com/article/10.3390/toxins13060376/s1, Figure S1: Tukey HSD tests for the effects of different carbohydrates and nitrogen sources, without interactions. Figure S2: Tukey HSD tests for the effects of different carbohydrates and nitrogen sources, with interactions. Figure S3: Tukey HSD test for the effect from sucrose levels and ammonium tartrate levels, without interactions. Figure S4: Tukey HSD test for the effect from sucrose levels and ammonium tartrate levels, including interactions. Figure S5: Tukey HSD test for the effect from sucrose levels and sodium nitrate levels, without interactions. Figure S6: Tukey HSD test for the effect from sucrose levels and sodium nitrate levels, with interactions. Figure S7: individual data for the four compounds, for the full factorial design experiment. Figure S8: Pigmentation of the media. Figure S9: Calibration curves. Table S1: Average yields of the media with different carbohydrates and the two types of nitrogen source. Table S2: Average yields of the media composed of three different levels of sucrose and three levels of ammonium tartrate. Table S3: Average yields of the media composed of three different levels of sucrose and three levels of sodium nitrate.
