# Supplementary material for: Production and Selectivity of Key Fusarubins from Fusarium solani due to Media Composition
Source: Toxins (Basel). 2021 May 25;13(6):376. doi: 10.3390/toxins13060376 (PMC8230112; doi:10.3390/toxins13060376)
Supplement: Supplementary file 1 [file toxins-13-00376-s001.zip › toxins-1204429-supplementary.pdf]

Supplementary Materials: Production and Selectivity of Key Fusarubins from *Fusarium solani* due to Media Composition

Sebastian Birkedal Kristensen, Tobias Bruun Pedersen, Mikkel Rank Nielsen, Reinhard Wimmer, Jens Muff and Jens Laurids Sørensen

Table S1. Average yields of the medias with different carbohydrates and the two types of nitrogen source.

	Ammonium Tartrate				Sodium Nitrate			
	**Maltose**	**Glucose**	**Sucrose**	**Glycerol**	**Maltose**	**Glucose**	**Sucrose**	**Glycerol**
Fusarubin	4.66	2.26	4.78	19.02	56.17	36.88	131.88	0.00
Javanicin	16.51	0.40	27.84	30.40	2.65	3.12	19.29	0.00
Bostrycoidin	175.76	2.34	30.52	39.71	1.69	1.19	1.62	0.00
Anhydrofusarubin	54.14	0.40	25.56	1.11	2.98	3.03	36.42	0.00

Table S2. Average yields of the medias composed of three different levels of sucrose and three levels of ammonium tartrate.

	**50g/L**			**100g/L**			**150g/L**		
	**4.6g/L**	**6.9g/L**	**9.2g/L**	**4.6g/L**	**6.9g/L**	**9.2g/L**	**4.6g/L**	**6.9g/L**	**9.2g/L**
Fusarubin	2.47	2.39	46.94	286.75	179.92	82.62	220.46	162.38	123.50
Javanicin	14.61	16.71	19.18	32.34	76.78	54.51	24.37	23.32	19.15
Bostrycoidin	256.49	198.13	198.26	195.35	184.05	179.15	158.06	112.67	164.82
Anhydrofusarubin	119.62	61.17	31.34	4.76	3.11	0.96	21.69	9.50	12.79

Table S3. Average yields of the medias composed of three different levels of sucrose and three levels of sodium nitrate.

	**50g/L**			**100g/L**			**150g/L**		
	**3g/L**	**4.5g/L**	**6g/L**	**3g/L**	**4.5g/L**	**6g/L**	**3g/L**	**4.5g/L**	**6g/L**
Fusarubin	75.32	89.63	130.00	90.85	117.49	115.95	61.67	72.19	63.56
Javanicin	19.28	27.86	27.94	21.00	33.49	30.36	46.16	49.12	32.98
Bostrycoidin	1.39	1.47	1.37	1.45	2.03	1.91	1.30	1.30	1.32
Anhydrofusarubin	30.88	26.93	14.13	9.58	9.60	7.36	4.67	8.29	7.63

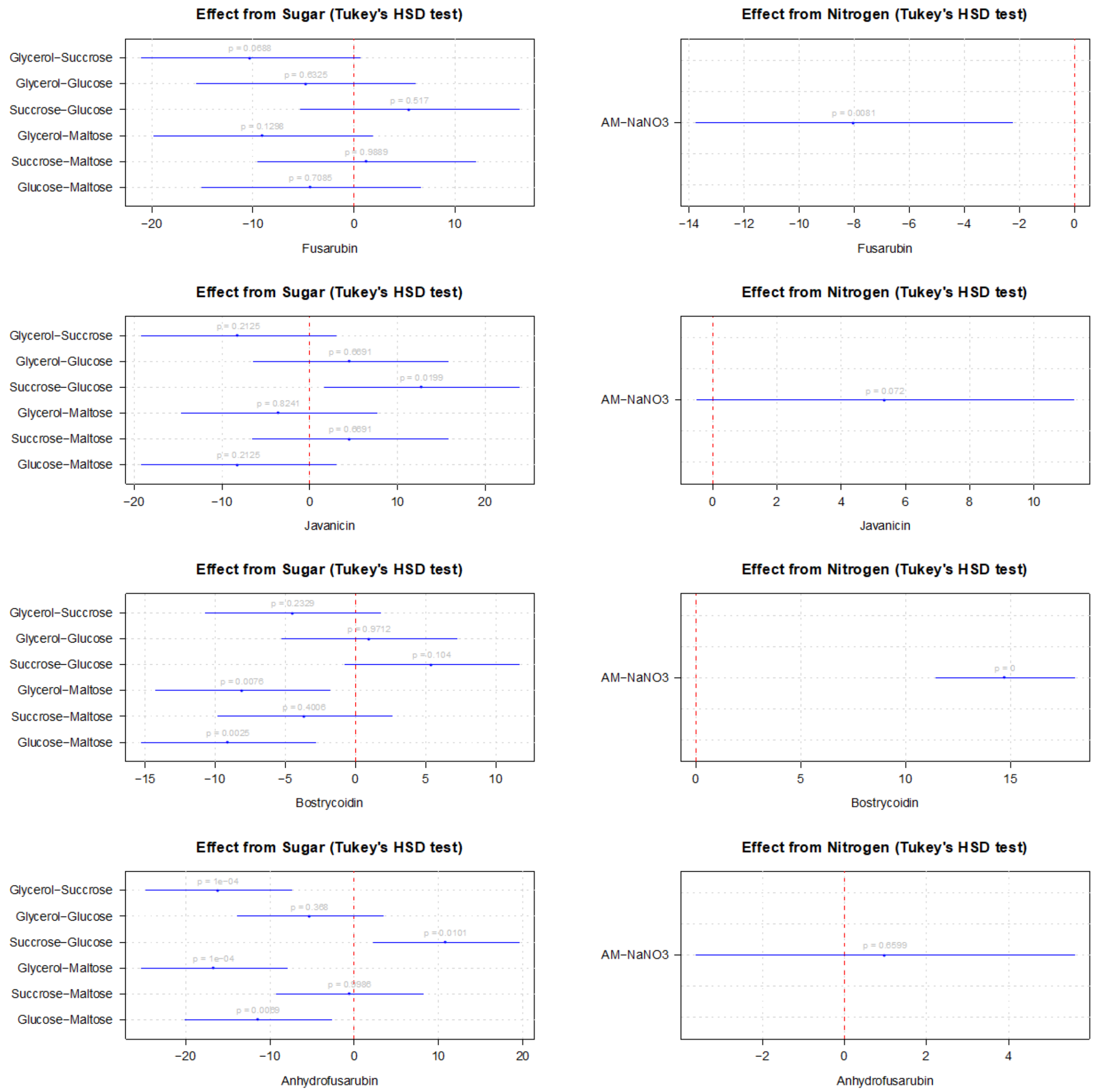

Figure S1. Tukey HSD tests for effects of different carbohydrates and nitrogen sources, without interactions.

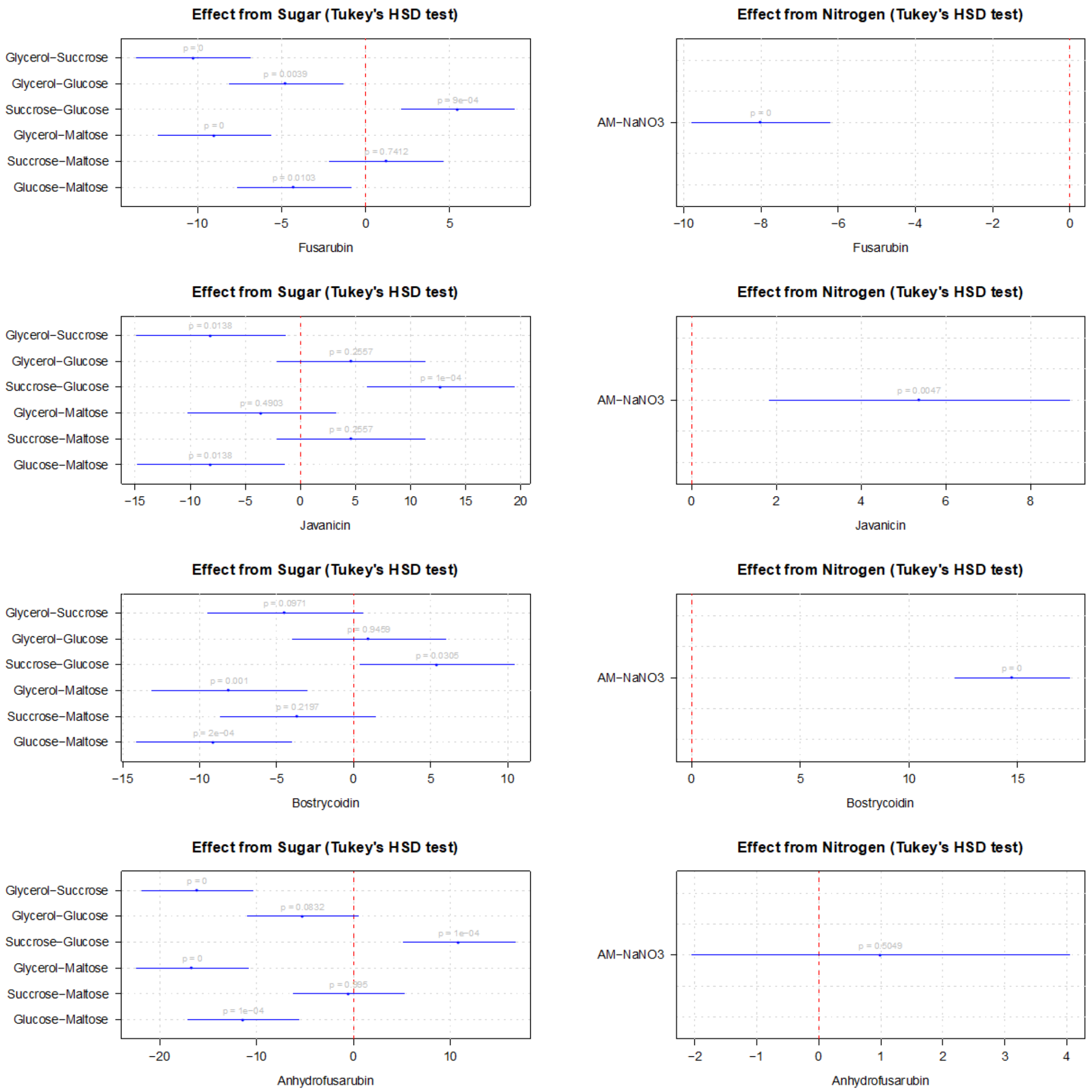

Figure S2. Tukey HSD tests for effects of different carbohydrates and nitrogen sources, with interactions.

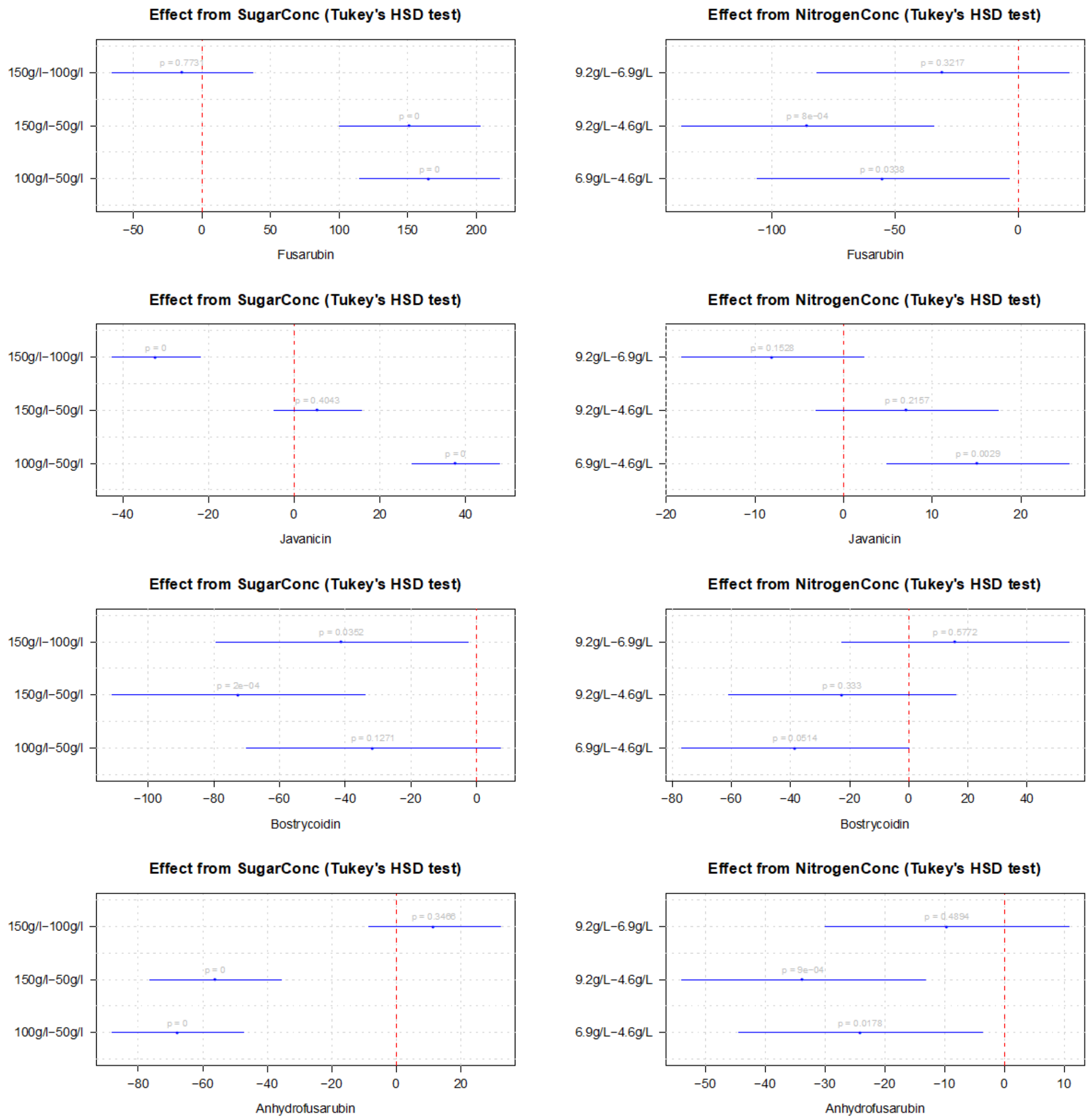

Figure S3. Tukey HSD test for effect from sucrose levels and ammonium tartrate levels, without interactions.

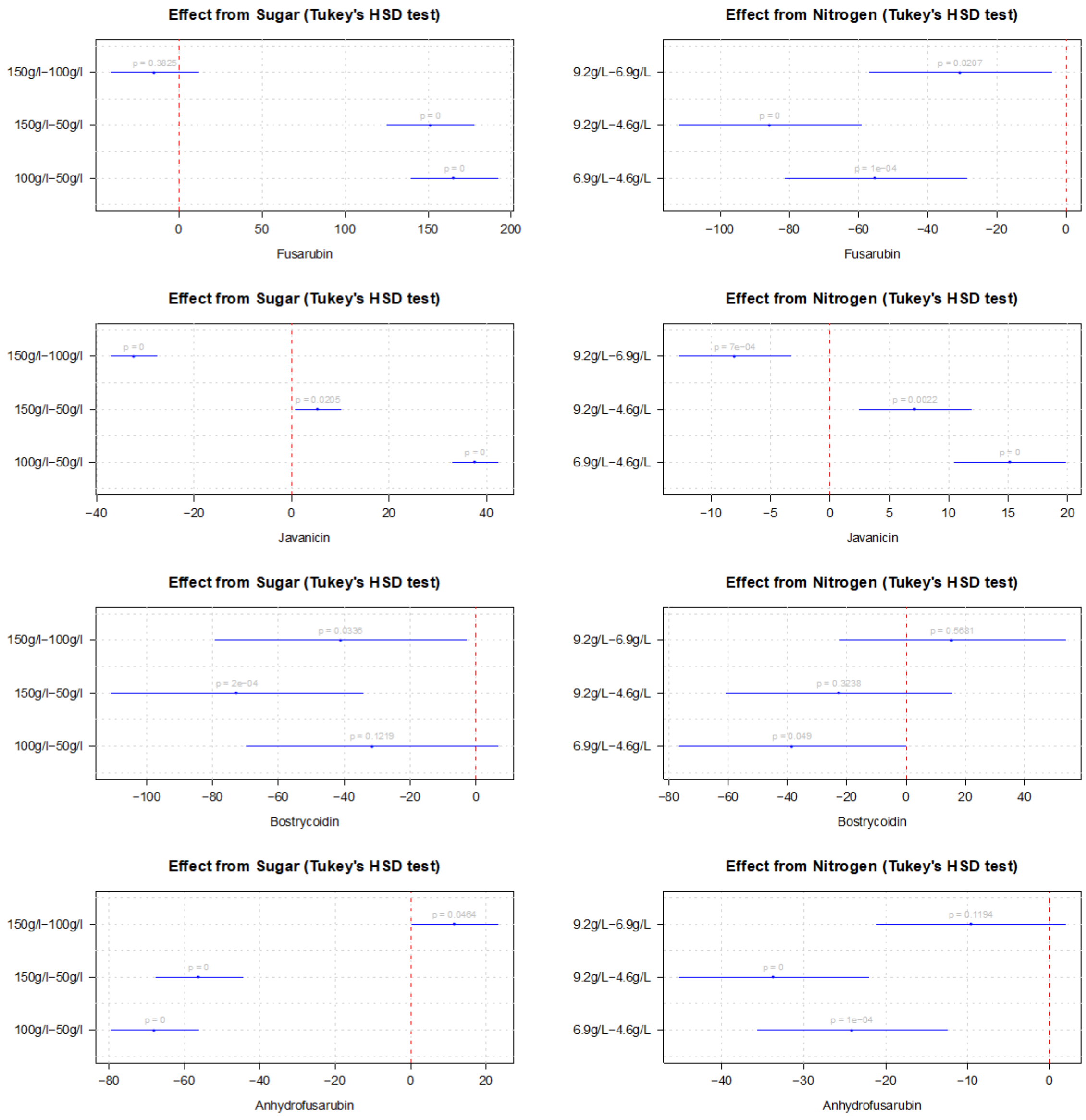

Figure S4. Tukey HSD test for effect from sucrose levels and ammonium tartrate levels, including interactions.

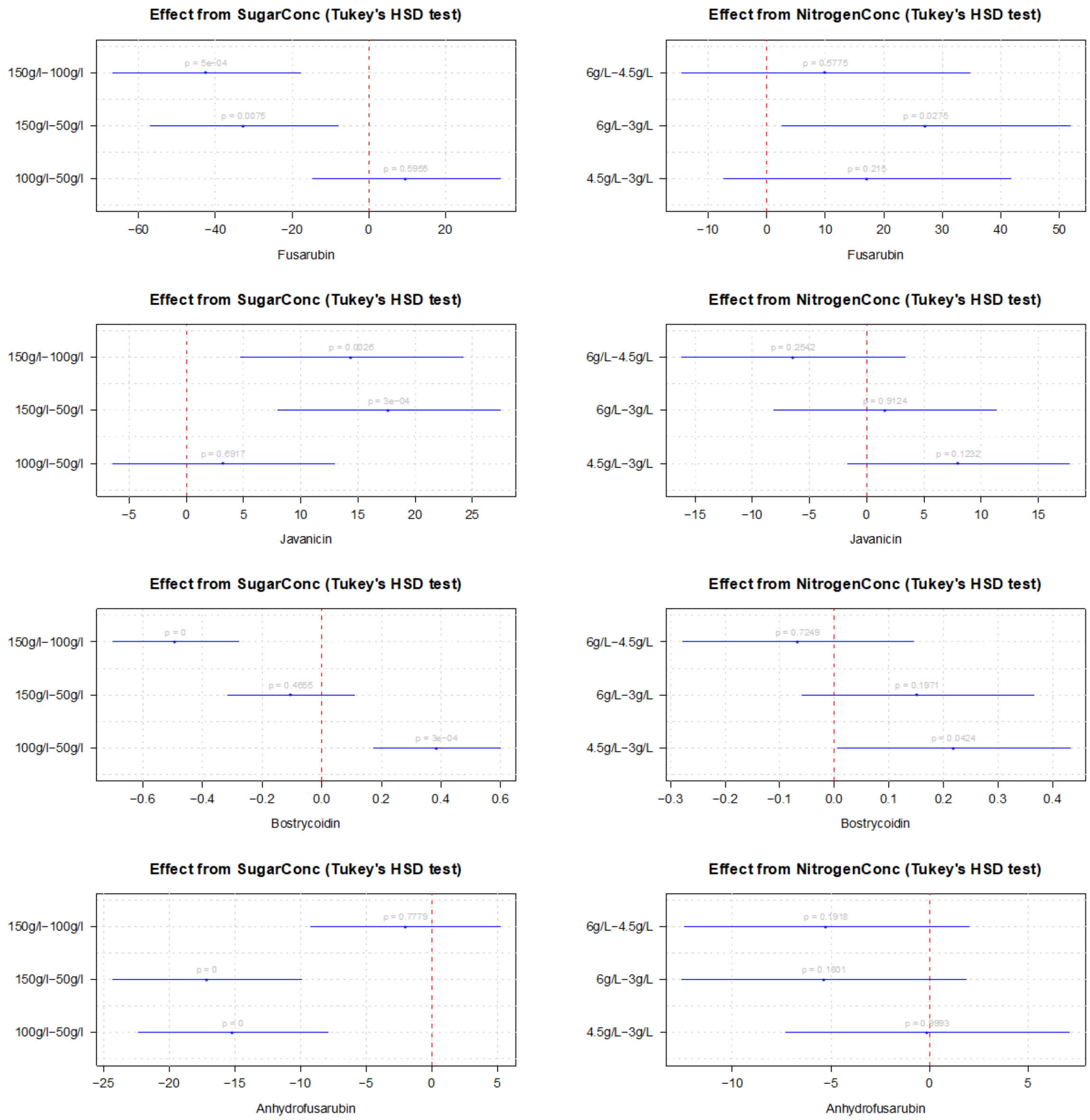

Figure S5. Tukey HSD test for effect from sucrose levels and sodium nitrate levels, without interactions.

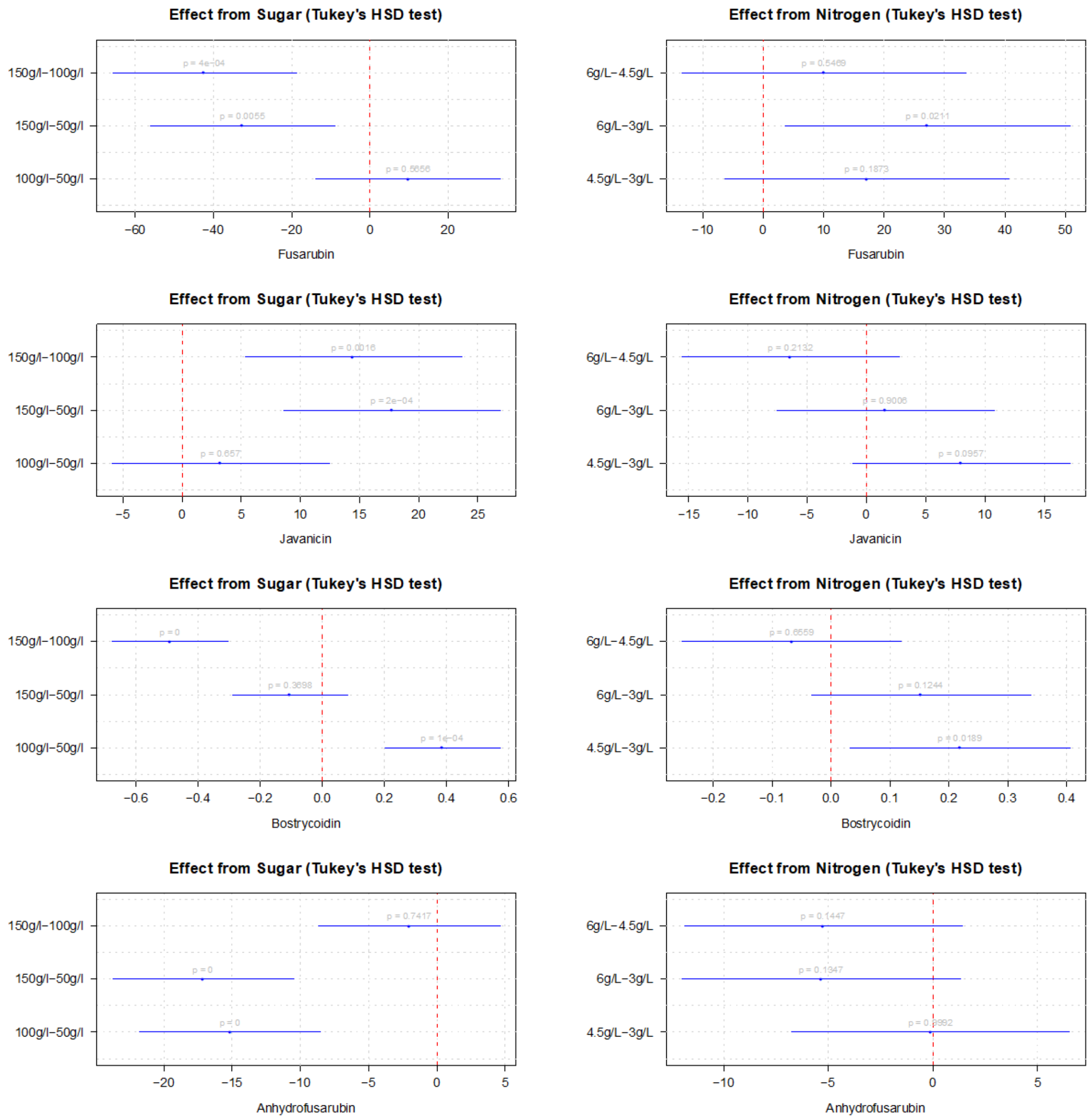

Figure S6. Tukey HSD test for effect from sucrose levels and sodium nitrate levels, with interactions.

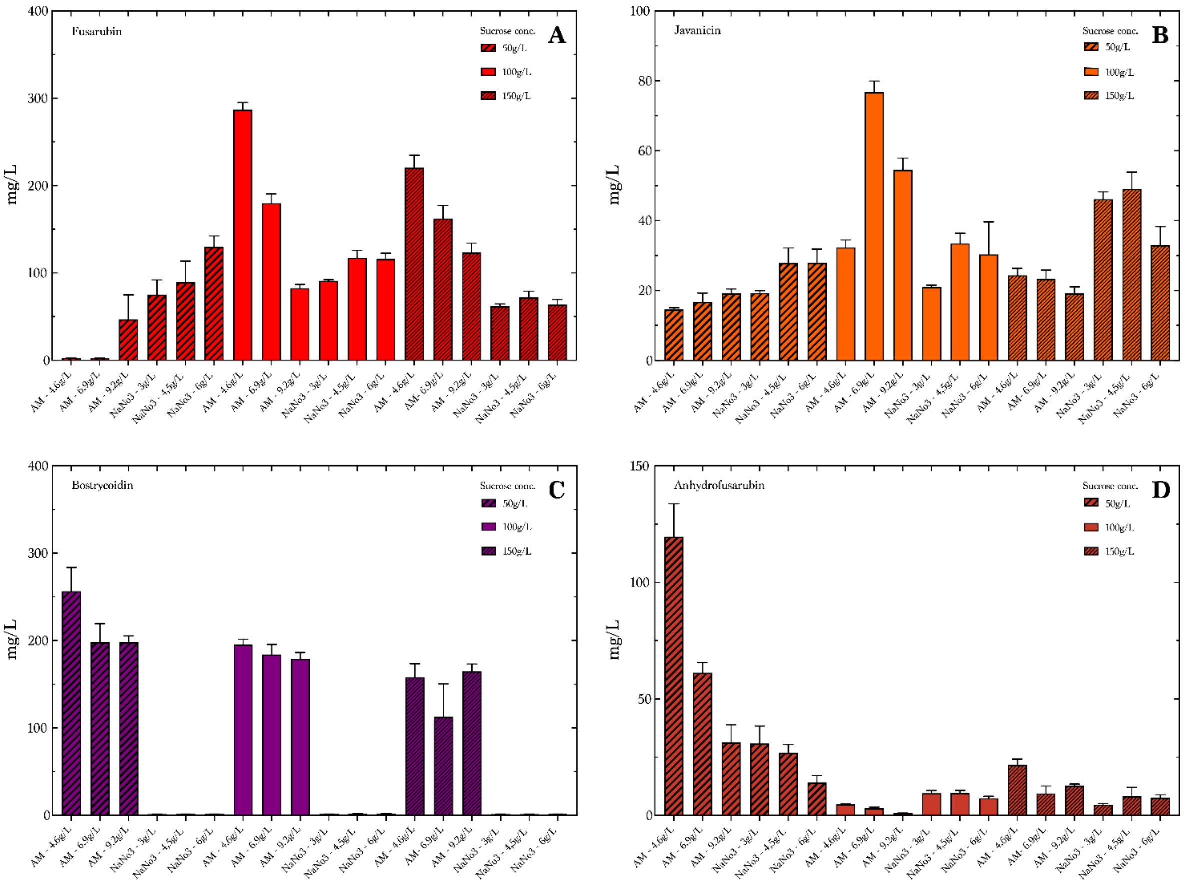

Figure S7. Individual data for the four compounds, for the full factorial design experiment, investigating sucrose as the carbohydrate and Ammonium tartrate and sodium nitrate as the nitrogen source. (**A**) Fusarubin, (**B**) Javanicin, (**C**) Bostrycoidin and (**D**) Anhydrofusarubin.

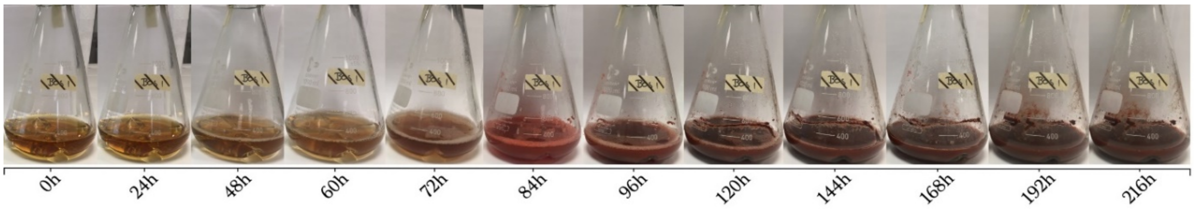

Figure S8. Pigmentation of the media, composed of 50g/L of maltose and 4.5g/L of ammonium tartrate, over time.

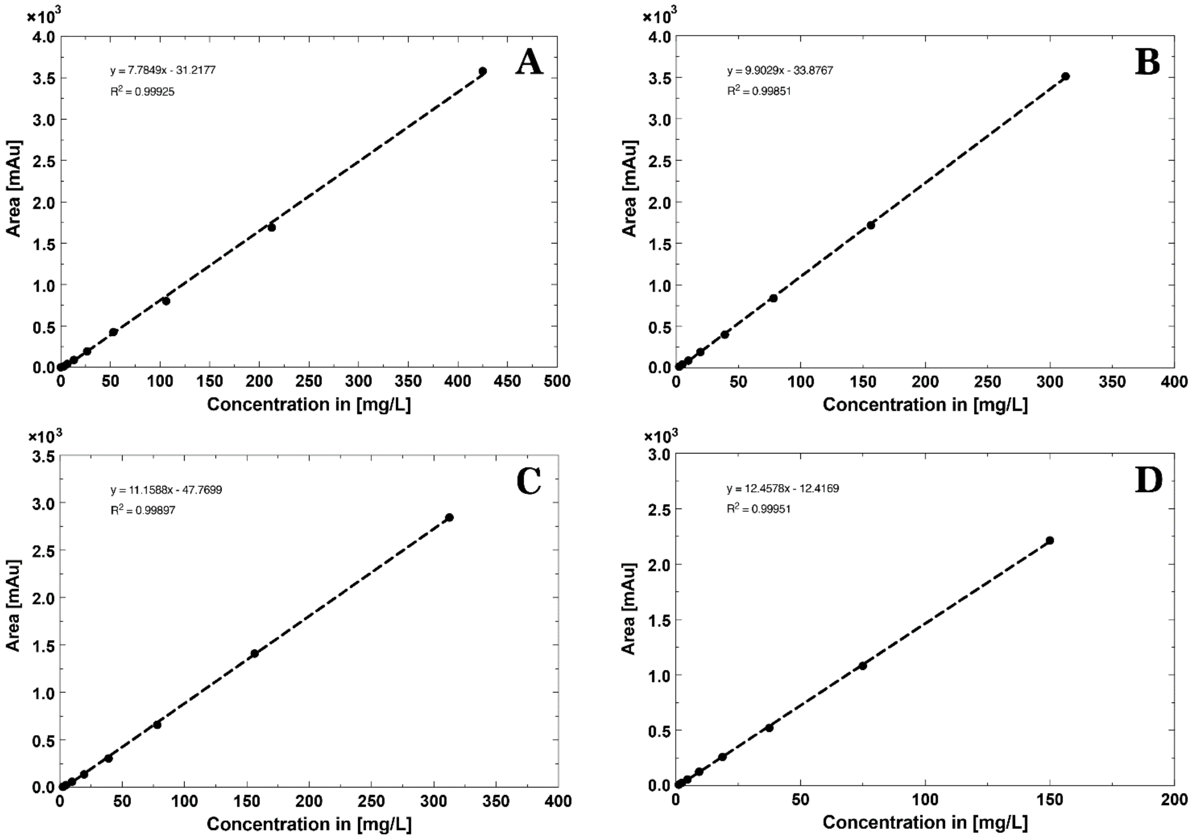

Figure S9. Calibration curves for the four compounds of interest in this study. (**A**) Fusarubin, (**B**) Javanicin, (**C**) Bostrycoidin and (**D**) Anhydrofusarubin.
